# Direct control of CSF pulsatility and its effect on CBF: initial findings using a novel method and device

**DOI:** 10.1186/1743-8454-7-S1-S12

**Published:** 2010-12-15

**Authors:** Stephen Dombrowski, Jun Yang, Serge El Khoury, Natalie Krajcir, Mark Luciano

**Affiliations:** 1 Section of Pediatric and Congenital Neurosurgery (S60), Department of Neurosurgery, Neurological Institute, Cleveland Clinic 9500 Euclid Avenue, Cleveland, OH 44195, USA

## Background

Over the past several years, there has been newly emerging and overlapping fields of research investigating CSF pressure and flow pulsatility (CSFp), and cerebral blood flow (CBF). It is not known whether CSFp is merely a passive epiphenomena or alternatively, plays a physiologically significant role in cerebral compliance and blood flow. Thus, the relationship between CSFp and CBF is not clearly understood. In order to study the effect of CSF pulsatility on blood flow, we have developed a novel method and device for controlling (i.e., reducing or augmenting) pulsatility.

## Materials and methods

In 15 canine subjects, a small (0.3-3.0cc), custom-made polyurethane bladder was surgically implanted in either the cranial or spinal epidural space such that it would alter the underlying CSF space. CSFp were controlled via an oscillating air pump gated to the cardiac cycle timed to deflate (ICP reduction) or inflate (ICP augmentation) during systole. Measures for ICP (fiberoptic probes) and CBF (laser-Doppler and thermal diffusion probes, microsphere injection, and SPECT-Tc-99) were obtained intra-operatively under different acute and chronic conditions.

## Results

CSPp reduction or augmentation was successfully achieved via cardiac-gated oscillation of a bladder device. Alteration of ICP-waveform was global and measured remotely on the contralateral side and with device activation in the spinal space. Under specific inflation cycles and physiological conditions, operation of the system increased CBF up to 15 mL/min*100gm, or by as much as 40%. Mean systemic pressure, cardiac output, and mean ICP did not significantly change with system activation.

## Conclusions

We have developed a method of altering CSF pressure pulsatility using cardiac-gated oscillating bladder which dynamically alters CSF space volume. The ability of the system to increase CBF without affecting cerebral perfusion pressure (MAP or ICP) suggests it may work through alteration of cerebrovascular compliance and impedance. Understanding the effect of CSFp on cranial compliance may ultimately allow a new therapy for increasing blood flow in acute and chronic states, including hydrocephalus, where CBF and CSFp are abnormal.

## Competing interests

SMD, MGL serve as Scientific Advisors for CSF Therapeutics, Inc.

